# Choice Between Salary and Employer Brand: The Roles of Materialism and Inclination to Develop an Identity-Motives-Based Relationship With an Employer Brand

**DOI:** 10.3389/fpsyg.2020.00555

**Published:** 2020-03-27

**Authors:** Małgorzata A. Styśko-Kunkowska, Zuzanna Kwinta

**Affiliations:** ^1^Department of Business Psychology and Social Innovations, Faculty of Psychology, University of Warsaw, Warsaw, Poland; ^2^Faculty of Psychology, University of Warsaw, Warsaw, Poland

**Keywords:** salary, employer brand, materialism, identity motives, job preferences, job evaluation, job satisfaction, life satisfaction

## Abstract

Despite recent interest in individual differences in psychological meanings of consumer brands, the concept of psychological employer brand as a factor independent of particular brands has not been examined. Drawing on an instrumental-symbolic framework, person–organization fit literature, and theory and research on salary and materialism, and combining consumer brand approaches with motivated identity construction theory, we examine the role of materialism and identity-motives-based inclination for the self–employer brand relationship in the situation of a dilemma between two job offers: one proposed by a strong employer brand with an unattractive salary and one from a subjectively weak brand with an attractive salary. A homogenous sample of 101 university students in academic fields related to financial careers participated in a quasi-experimental study. We found that participants preferred the offer from the weak employer brand with an attractive salary compared to the strong employer brand with an unattractive salary; however, supporting our hypothesis, those who preferred this offer anticipated lower job satisfaction. Following expectations, materialism negatively and inclination for self–employer brand relationship positively predicted preferences and evaluations of the unattractive salary offer proposed by the strong employer brand. However, materialism negatively predicted anticipated job satisfaction regarding this offer, as well as positively predicting evaluation of the weak brand with attractive salary job offer. Despite all the detailed hypotheses not being supported, the findings confirm the role of materialism in job offer preference and introduce the inclination to develop an identity-motives-based relationship with an employer brand as an important factor in reactions toward different employer branding recruitment strategies. We discuss the results in light of previous theories and research on person–organization fit, materialism, and brand effects, and consider potential short- and long-term outcomes of recruitment strategies.

## Introduction

When searching for a job, potential employees may face a dilemma of which job offer to choose: one with a high salary proposed by an organization perceived as having a poor employer image, or one with an unattractive salary offered by a company evaluated as having a strong employer brand. Business representatives and academics consider the latter strategy – reducing compensation for companies with high levels of firm reputation – as one of the effective recruitment approaches (Schlager et al., 2011, pp. 497–508), while young adults may be particularly interested in higher salary as a career starting point. In the present study, we aimed to identify what drives preferences and evaluations of such job offers by investigating the impact of materialism and of a new construct of inclination to develop identity-motives-based relationships with employer brands.

In 1994, Cable and Judge were the first to empirically confirm widespread beliefs about preferences for job offers with high salaries, identifying materialism as one of the important underlying determinants. Their findings were supported by further academic and business research (e.g., Chapman et al., 2005, pp. 928–944; Uggerslev et al., 2012, pp. 597–660). For example, in an international survey conducted in emerging and developed countries with a sample of adults born after 1982, Deloitte (2016) demonstrated that pay and other financial incentives were the most important factors for potential employees when choosing an organization, followed by other considerations such as work-life balance, advancement opportunities, flexibility, and a sense of meaning.

Previous research has indicated that perception, evaluation, and attitudes toward organizations also constitute key determinants of organization attractiveness (Chapman et al., 2005, pp. 928–944), intent to apply to a given organization and application decisions (Collins and Stevens, 2002, pp. 1121–1133), and job acceptance (Powell, 1991, pp. 67–83; Powell and Goulet, 1996, pp. 1619–1640). Furthermore, studies have shown that reputation and first impressions of an organization impacted decisions on participating in further steps of the recruitment process (Turban and Cable, 2003, pp. 733–751), and directly influenced the number and quality of applicants (Collins and Han, 2004, pp. 685–717).

Although both salary and perception of an employer brand constitute important criteria for the attractiveness of an organization, their relative importance is contextual (e.g., Tom, 1971, pp. 573–592; Cable and Judge, 1994, pp. 217–248; Cable and Turban, 2001; Turban and Cable, 2003, pp. 733–751; Chapman et al., 2005, pp. 928–944; Lievens, 2007, pp. 51–69; Lauzier and Roy, 2011, pp. 35–46; Van Hoye and Saks, 2011, pp. 311–335; Franca and Pahor, 2012, pp. 78–122; Uggerslev et al., 2012, pp. 597–660). Research using an instrumental-symbolic framework (Lievens and Highhouse, 2003, 75–102) revealed that the impact of instrumental organization features, including salary, and of symbolic ones (e.g., personality characteristics attributed to the organization) differed among business sectors (Lievens and Highhouse, 2003, pp. 75–102), target groups (prospective applicants, actual candidates, their companions, or employees of competitive organizations; Lievens et al., 2005, pp. 553–572; Lievens, 2007, pp. 51–69) and also depended on gender and age (Sutherland et al., 2002, pp. 13–20) and personality traits (Schreurs et al., 2009, pp. 35–46; Slaughter and Greguras, 2009, pp. 1–18). The relative roles of instrumental and symbolic features also varied by outcomes, such as attractiveness, identification, or satisfaction (Lievens et al., 2007, pp. S45–S59; Priyadarshi, 2011, pp. 510–522). Uggerslev et al. (2012, pp. 597–660) found that organizational features (e.g., image, prestige, development, challenge, advancement/promotions, autonomy, employee relations/treatment, coworkers, and teamwork/social activities) were more important in the early stages of the process, while job characteristics, including salary, were important in all stages.

Meta-analyses revealed that the perception of fit between a potential employee and job or organization was even more important than salary and organization image in predicting outcomes, including job pursuit intentions, job/organization attraction, acceptance intentions, and job choice (Chapman et al., 2005, pp. 928–944; Uggerslev et al., 2012, pp. 597–660). Implementing a person–organization (P-O) fit approach, Cable and Judge (1994, pp. 294–311) found that materialists placed higher importance on salary than other people. They presumed that higher salary satisfies materialists’ needs particularly well. Similarly, Richins and Dawson (1992, pp. 303–316) revealed that materialists express a greater need for financial security and consider a high annual household income necessary to satisfy their needs. Materialists attach a higher value to material goods, ascribe a central role to them in their lives, and acquire them as part of their pursuit of happiness guiding their choices and behaviors. They value material goods because they perceive them as having high financial value, being status symbols, and for their appearance, as well as for utilitarian reasons (Richins and Dawson, 1992, pp. 303–316; Richins, 2004, pp. 209–219).

We applied a P-O fit approach to understand individual preferences for strong employer brands (EBs). We posit that for some people, EBs may have particular value. First, studies on consumer brands confirm important psychological roles brands have in individual, group and social identity construction, enhancement, and expression (Park et al., 1986, pp. 135–145; Gardner and Levy, 1955, 33–39; Pham, 1998, 144–159; Escalas and Bettman, 2005, pp. 378–389; Tietje and Brunel, 2005, 135–154; Kapferer, 2008; Keller, 2008; Strizhakova et al., 2008, pp. 82–93; Park et al., 2008; Aaker, 2009; Parks and Guay, 2009, pp. 675–684; Reimann and Aron, 2009, pp. 65–81; Fischer et al., 2010, pp. 9–34). Second, in recent years, research has shown individual differences in cognitive and motivational-affective meanings of brands, which have an impact on processing information and preferences (Sprott et al., 2009, pp. 92–104; Puligadda et al., 2012, pp. 115–130). For example, Sprott et al. (2009, pp. 92–104) described the individual tendency to consider brands as important to one’s self-concept, known as brand engagement in self-concept (BESC), and showed that BESC is related to giving more attention to, better recalling, and more preferring their favorite brands and having better memories of those brands, as well as less sensitivity to the price of preferred brands. Flynn et al. (2011, pp. 5-18) found that BESC correlated negatively with age and positively with household income, but was independent of gender and education level. Other studies have shown that EBs may have similar psychological meanings to those attached to consumer-branded products. The role of self-perception has long been a topic of discussion in literature on P-O fit (Tom, 1971, pp. 573–592; Kristof, 1996, pp. 1-49). Individual identity relates to job identity (Blustein et al., 1989, pp. 196–202; Côté, 2002, pp. 117–134), and research has provided evidence for identity importance in job attractiveness (Highhouse et al., 2007, pp. 134–146) and organizational identification (Thomas et al., 2017, pp. 508–523). Work constitutes part of the material self (Tian and Belk, 2005, pp. 297–310), and people often spend a lot of their time working (Czapiński, 2015). Additionally, Nolan and Harold (2010, pp. 645–662) revealed that how well a person’s actual image and, to a lesser extent, ideal image, fit an organization was a significant predictor of organizational attractiveness.

By combining consumer brand models with the motivated identity construction theory (MICT, Vignoles et al., 2006, pp. 308–333; Vignoles, 2011, pp. 403–432), the first author conceptualized a model of the inclination to develop a psychological relationship between self and EB based on identity motives (self–employer brand relationship; S-EB-R). It follows the assumptions of the MICT that identity refers to all facets of the mental representation of the self and may affect individuals’ thoughts, feelings, and actions. Self-esteem, continuity, distinctiveness, meaning, efficacy, and belonging constitute six main identity motives. According to the S-EB-R model, some people are more inclined to have positive emotions, beliefs, and behavioral intentions toward EBs, which could satisfy their identity motives, showing a higher tendency to develop a psychological relationship between the self and EBs. In line with brand models, strong brands are predisposed to satisfy not only functional needs but also psychological and identity needs (Kapferer, 2008; Keller, 2008). Thus, strong brands may be particularly valuable for people with higher inclinations to build relationships between themselves and EBs.

Previous research has posited that salary and materialism, as well as employer brand name, identity motives, and satisfaction by EBs, may influence not only preferences, evaluations, and perceptions of fit but also beliefs about job offer satisfaction (Richins and Dawson, 1992, pp. 303–316; Unanue et al., 2016, pp. 10–22; Unanue et al., 2017b, p. 1755; Wang et al., 2017, pp. 312–317). Money (as an example of an extrinsic motivator; Unanue et al., 2016, pp. 10–22) and materialistic values were found to be related to lower satisfaction with life overall and with specific areas of life, including income and standard of living (Richins and Dawson, 1992, pp. 303–316), with lack of satisfaction and frustration of basic psychological needs as mediators (Unanue et al., 2017b, p. 1755; Wang et al., 2017, pp. 312–317). However, the direction of this relationship may depend on the wealth of a country (Zawadzka et al., 2018, pp. 1–14) and on country-specific work-role outputs, including salaries (Sousa-Poza and Sousa-Poza, 2000, pp. 517–538). In turn, indirect arguments for the positive relationship of identity motives and satisfaction can be found in literature on needs satisfaction (Vignoles, 2011, pp. 403–432; Fisher, 2014, pp. 9–34; Unanue et al., 2014, pp. 569–585; Greenaway et al., 2015, pp. 294–307; Unanue et al., 2017a, p. 1755), identity (Oyserman and James, 2011, pp. 116–145), P-O fit (Kristof, 1996, 1–49; Gabriel et al., 2014, 389–420) and strong brands (e.g., Keller, 2008). Directly, research also confirmed the link between EBs perception and job satisfaction (Davies, 2008, pp. 667–681; Schlager et al., 2011, pp. 497–508; Styśko-Kunkowska, 2017, pp. 65–84). Additionally, Sprott et al. (2009, pp. 92–104) claimed that there is no reason to expect relationships of BESC with global assessments of the self and evaluation of overall life satisfaction, as they are separate constructs.

Thus, some people may attempt to satisfy their desire for material goods through a variety of means, including salary. Furthermore, for some individuals, brands constitute an essential means to construct and express their multifaceted identities, and brands and identity motives may drive preferences and evaluations. However, the existence and nature of impact of identity-based inclinations to develop relationships with EBs based on preferences and more positive evaluations of strong brands juxtaposed with unattractive job features such as low salary, has not yet been empirically proven.

In the present study, we examine how placing material goods at the center of one’s life and a new construct of identity-motivated inclination to develop a psychological relationship with EBs predicts preferences, evaluations, and anticipated job satisfaction in a dilemma between two job offers, which vary in terms of salary attractiveness and perceived EB equities.

Hypothesis 1: Materialism will influence preferences and evaluations of job offers and will be related to life satisfaction.

• Participants with higher levels of materialism will prefer the job offer with the attractive salary proposed by the weak EB (weak employer brand, attractive salary: WEBAS offer) over the job offer with the unattractive salary proposed by the strong EB (strong employer brand, unattractive salary: SEBUAS offer) (H1a).
• The higher the level of materialism, the more positive the evaluation of the WEBAS offer (H1b), less positive the evaluation of the SEBUAS offer (H1c), higher the subjective sense of fit toward the organization with the WEBAS offer (H1d), lower the subjective sense of fit toward the organization with the SEBUAS offer (H1e), higher the anticipated job satisfaction with the WEBAS offer (H1f), lower the anticipated job satisfaction with the SEBUAS offer (H1g), and lower the sense of overall life satisfaction (H1h).

Hypothesis 2: Subjective psychological EB meaning will influence preferences for and evaluations of job offers.

• Participants with a higher level of inclination for S-EB-R will prefer the SEBUAS offer compared to the WEBAS offer (H2a).
• The higher the level of inclination for S-EB-R, the more positive the evaluation of the SEBUAS offer (H2b), less positive the evaluation of the WEBAS offer (H2c), higher the subjective sense of fit toward the organization with the SEBUAS offer (H2d), lower the subjective sense of fit toward the organization with the WEBAS offer (H2e), higher the anticipated job satisfaction with the SEBUAS offer (H2f), and lower the anticipated job satisfaction with the WEBAS offer (H2g).

Hypothesis 3: Supporters of the WEBAS offer will reveal lower anticipated job satisfaction as compared to supporters of the SEBUAS offer. We do not expect significant relationships between preferences and overall life satisfaction.

## Materials and Methods

To test our hypotheses, we conducted a quasi-experimental study with two between subject independent variables and several within subject measures of dependent variables. The present study was preceded by a pilot study to prepare stimuli material (see Supplementary Material).

### Participants

Participants were 101 university students (66.3% women) in the fields of management, economics, and mathematical sciences from 15 public and private universities in Poland. Their ages ranged between 20 and 38 years (*M* = 21.83; *SD* = 2.16). In total, 74.3% of the participants reported having work experience with at least one employer; 47.5% reported they worked in the past 2–30 months, mainly in private companies (93.8%); and a 21.8% reported they planned to engage in a job search within the next 3 months. We aimed for homogeneity in participants’ fields of study to maximize the probability of shared sets of considered EB and job positions, and shared perception of brands’ equity and the attractiveness levels of salaries. To establish sample size we followed the assumptions about that minimum sample size of 15 cases for predictor in regression (Field, 2013) and at least 100 of participants in logistic regression (Long, 1997). The calculated sample size for regression with expected effect size of 0.2 and two predictors was 81 (calculated with GPower).

### Procedure

After administering screening questions about being a student and field of study, we measured levels of materialism and inclination for EB relationships, and assessed reactions toward two job offers, including offer preferences and evaluations. Before these evaluations, we measured participants’ levels of overall life satisfaction and anticipated job satisfaction regarding a chosen offer. Additionally, we assessed the effectiveness of the manipulation, controlled for the level of familiarity with the companies, and gathered personal, educational, and job-related data. We used the Computer-Assisted Web Interview (CAWI) on the LimeSurvey platform, and distributed a link on online forums for students of management and economic sciences, similar to the pilot study (see Supplementary Material). The design of the study was approved by the Committee of Ethics in Research of the Faculty of Psychology, University of Warsaw.

### Stimuli Material and Tools

#### Materialism

To measure participants’ level of materialism, we used the Polish Scale of Attitudes toward Material Goods (AMG; Górnik-Durose, 2002), which is based on the Material Values Scale (MVS) by Richins and Dawson (1992, pp. 303–316). Cronbach’s alpha for the one-factor scale, including all items, was 0.90. Thus, as in the original version, we composed an indicator as the mean of ratings for all the items. We present descriptive statistics in Table 1.

**TABLE 1:** Descriptive statistics for materialism, inclination for S-EB-R, and job offer evaluations.

	Type of job offer	Mean	*SD*	*N*
Materialism		4.04	0.94	101
Inclination for S-EB-R		3.33	0.52	101
**Employer brand equity**				
Supporters of SEBUAS	SEBUAS	5.56	0.97	36
	WEBAS	3.13	0.95	36
Supporters of WEBAS	SEBUAS	4.93	0.84	65
	WEBAS	3.90	0.76	65
**Attractiveness of salary**				
Supporters of SEBUAS	SEBUAS	2.39	0.90	36
	WEBAS	4.39	0.64	36
Supporters of WEBAS	SEBUAS	2.06	1.07	65
	WEBAS	4.51	0.56	65
**Attractiveness of job offer**				
Supporters of SEBUAS	SEBUAS	5.51	1.01	36
	WEBAS	3.54	1.12	36
Supporters of WEBAS	SEBUAS	4.46	1.22	65
	WEBAS	4.84	1.09	65
**Perception of organizational fit**				
Supporters of SEBUAS	SEBUAS	5.44	0.86	36
	WEBAS	2.65	0.87	36
Supporters of WEBAS	SEBUAS	4.44	1.11	65
	WEBAS	3.62	0.83	65

*S-EB-R, self–employer brand relationship; SEBUAS, job offer from a strong employer brand with an unattractive salary; WEBAS, job offer from a weak employer brand with an attractive salary.*

#### EB Psychological Meaning

To measure EB psychological meaning, we used the Inclination for Self–employer Brand-Relationship scale (Incl-S-EB-R). This questionnaire measures the inclination to satisfy identity motives through EB overall, as well as to satisfy 15 identity sub-motives separately (2–3 sub-motives each for Belonging, Continuity, Distinctiveness, Efficacy, Meaning, and Self-Esteem) or grouped as compensational or expressive. The measure includes cognitive, affective, and behavioral components. Examples of items are “I would consider only offers from employers who care about harmonious teams” (Good atmosphere self-expressive sub-motive of the Belonging behavioral component) or “The prestigious brand of my employer would make me feel superior to other people” (Superiority compensational sub-motive of Distinctiveness cognitive component). The Incl-S-EB-R scale was constructed and piloted qualitatively and quantitatively. In the present study, we applied the Incl-S-EB-R-3 version in which participants rate 101 statements on a scale ranging from 1 “it fits me very weakly” to 5 “it fits me very strongly.” For this study, we used an overall index constructed based on the mean for all 101 items, with higher scores indicating stronger identity-motives-based inclination to develop self-EB relationships. Cronbach’s alpha coefficient for this index was 0.97. We present descriptive statistics in Table 1.

#### Job Offers

The SEBUAS job offer was ascribed to Google and offered a gross salary of 2,500 in local currency (Polish złoty). The WEBAS job offer was from McDonald’s, with a gross salary of 6,000 in local currency. Brand names, salary amounts, and job titles were chosen based on a pilot study conducted with a similar target group as the present study (see Supplementary Material). Considering the complexity of students’ job preferences, job offers differed only by brand names and salaries. To maintain a transparent experimental design, we instructed participants to imagine that they were choosing their first job after graduation, and two companies proposed similar job offers for the position of financial controller with a similar range of duties, but that the job offers differed in terms of salaries. We presented the two offers on a table, in counterbalanced order.

#### Offer Preferences

We measured the main dependent variable, offer preferences, with one dichotomous question, “Which offer would you choose?” with response choices of “Offer A” or “Offer B.”

#### Offer Evaluation

We measured offer attractiveness and subjective organizational fit. The former included two items about the employer (“In my opinion this employer is attractive” and “I really would like to participate in a recruitment process for this employer”) and two items about the offer itself (“This offer is attractive to me” and “I would like to get more information about this offer”). To measure organizational fit, we used items from the subjective sense of P-O fit questionnaire constructed by Czarnota-Bojarska (2006, pp. 151–161). Organizational fit included complementary fit (“The work in this company corresponds to my abilities”), supplementary fit (“I fit this company”), and organizational identification (“I identify with this organization”). All items were measured on a scale ranging from 1 “I strongly disagree” to 7 “I strongly agree.” Cronbach’s alpha coefficients exceeded 0.83. We used means for items to construct the indicators.

#### Anticipated Job Satisfaction and Overall Life Satisfaction

To measure anticipated job satisfaction related to a preferred offer, we used one question, “In your opinion, to what extent would you be satisfied with this job?” with a scale ranging from 1 “I would not be satisfied at all” to 5 “I would be strongly satisfied.” To ascertain that our measure assessed the attitude toward the offer, we controlled for overall life satisfaction level using the Satisfaction with Life Scale (SWLS) of Diener et al. (1985, pp. 71–75), including five items measured on a 5-point Likert-type scale, with anchors of 1 “disagree” and 5 “agree.” Higher scores on the SWLS indicated higher life satisfaction. Cronbach’s alpha was 0.885.

#### Manipulation Effectiveness

To check whether participants differentiated between the strength of the presented EBs, we used the same four items as in the pilot study (see Supplementary Material) comprising the Employer Brand Equity scale (EBE-2s). Items referred to perceptions of employees’ satisfaction, organization reputation as an employer, opportunities for employees’ development, and a sense of security at the organization. The same 7-point scale was applied as for offer evaluation. A principal components analysis with oblimin rotation revealed a one-factor structure. Cronbach’s alpha was 0.89 for the Google brand and 0.83 for the McDonald’s brand. Higher mean scores indicated a strong brand and lower scores a weak brand, for all four items. In the study, we applied one separate item to measure brand familiarity, “The company is well known,” with the measurement identical to offer and brand evaluation. Most brand models (see Keller, 2008) consider brand familiarity as a basic level of brand equity. We decided to treat this variable autonomously for three reasons. First, associations with unfamiliar brands are based only on name connotations, and thus do not activate any strong functional nor emotional associations. Second, the probability in the real world that people would choose an offer from an unknown institution without gathering additional information is very low; thus, the floor effect could appear. Third, some studies report that brand familiarity is less valued by students compared to other EB image features (Arachchige and Robertson, 2011, pp. 25–46).

For each job offer, we asked whether the salary offer was attractive, with answers on a 5-point scale ranging from 1 “definitely unattractive” to 5 “definitely attractive.” Additionally, for each offer, we asked one question about the attractiveness of the job position, using the same 5-point scale to confirm comparability of offers.

### Model of Analysis

To check effectiveness of manipulation and to understand the patterns of relationships between the preferences and evaluations, we conducted the series of the mixed design ANOVA. To analyze the frequency of preferences, we conducted χ^2^ analysis. To verify hypotheses H1a and H2a, we used logistic regression analysis. To analyze the results regarding H1b-H1h and H2b-H2g, linear hierarchical regression was applied. We tested Hypothesis 3 with a *t*-test for independent samples. Whenever we present bootstrap procedures, we report 95% BCA bootstrap confidence intervals based on 1,000 samples.

## Results

### Manipulation Check

To check the effectiveness of the manipulation, we conducted a series of mixed ANOVAs, in which we compared brand strength and perception of salary attractiveness between offers. We present descriptive statistics in Table 1 and results of the analysis in Table 2. We found significant large main effects of manipulation and weaker interactions. On average, participants ascribed significantly higher ratings on the EBE-2s questionnaire to Google (*M* = 5.16, *SD* = 0.93) than McDonald’s (*M* = 3.63, *SD* = 0.91). The interaction shows that differences between means for EBs were significant for supporters of each offer type, although supporters of the WEBAS offer perceived a smaller difference (*diff* = 1.035) between brand equities of the respective EBs [*F*(1,99) = 47.69, *p* < 0.001, $\eta_{\text{p}}^{2}$ = 0.325], than supporters of the SEBUAS offer [diff = 2.43; *F*(1,99) = 15.77, *p* < 0.001, $\eta_{\text{p}}^{2}$ = 0.60]. Differences between both sets of supporters were significant for strong EB [*diff* = 0.63; *F*(1,99) = 11.62, *p* < 0.001, $\eta_{\text{p}}^{2}$ = 0.105] and weak EB [*diff* = −0.77, *F*(1,99) = 19.74, *p* < 0.001, $\eta_{\text{p}}^{2}$ = 0.17]. Additionally, both companies were perceived as well known (both means exceeded 6). The difference in brand familiarity between Google (*M* = 6.35, *SD* = 0.79) and McDonald’s (*M* = 6.18, *SD* = 0.89) was small (0.17, BCa 95% CI [0.02, 0.33]), significant [*t*(100) = 2.02, *p* = 0.046], and represented a small-sized effect (*d* = 0.20).

**TABLE 2:** Mixed-design ANOVA with job offer preferences as a between-subject factor and offer type as a within-subject factor.

Source	df	MS	*F*	*p*	Effect size
**Employer brand equity**					
Type of job offer	1	139.10	190.68	0.00	0.66
Type of job offer × job offer preference	1	22.57	30.94	0.00	0.24
Error	99	0.73			
Job offer preference	1	0.23	0.30	0.58	0.00
Error	99	0.75			
**Attractiveness of salary**					
Type of job offer	1	229.00	404.62	0.00	0.80
Type of job offer × job offer preference	1	2.31	4.07	0.05	0.04
Error	99	0.57			
Job offer preference	1	0.50	0.62	0.43	0.01
Error	99	0.82			
**Attractiveness of job offer**					
Type of job offer	1	29.20	23.35	0.00	0.19
Type of job offer × job offer preference	1	64.35	51.45	0.00	0.34
Error	99	1.25			
Job offer preference	1	0.69	0.54	0.46	0.01
Error	99	1.28			
**Perception of organizational fit**					
Type of job offer	1	150.72	172.15	0.00	0.63
Type of job offer × job offer preference	1	45.24	51.68	0.00	0.34
Error	99	0.88			
Job offer preference	1	0.01	0.01	0.94	0.00
Error	99	0.88			

*MS = Mean squares, effect size = $\eta_{\text{p}}^{2}$.*

Regarding salary attractiveness, participants perceived the McDonald’s offer as significantly more attractive (M = 4.47, *SD* = 0.59) than the Google offer (M = 2.18, *SD* = 1.02). This difference represented a large-sized effect, particularly compared to the much smaller interaction effect. We confirmed the significance of differences both for supporters for the SEBUAS offer [F(1,99) = 127.22, *p* = 0.001, $\eta_{\text{p}}^{2}$ = 0.56] and the WEBAS offer [*F*(1,99) = 343.605, *p* < 0.001, $\eta_{\text{p}}^{2}$ = 0.78]. Differences between sets of supporters within types of offer were not significant (*p*s > 0.05).

To exclude differences in perception of job position, we compared the difference in attractiveness of job position between the SEBUAS (*M* = 3.30, *SD* = 1.10) and WEBAS offers (*M* = 3.25, *SD* = 1.10). Following our expectations, this difference (0.05, BCa 95% CI [−0.01, 1.10]) was not significant [*t*(100) = 1.52, *p* = 0.13, *d* = 0.15].

Based on these results, we concluded that both manipulations were effective.

### Job Offer Preferences and Evaluations

Before checking for evidentiary support for the hypotheses, we analyzed the frequency of job offer preferences and the relationship of preferences with evaluations. We found that a minority of participants (35.6%) preferred the SEBUAS offer, whereas a majority (64.4%) preferred the WEBAS offer. We confirmed the significance of the difference by chi-squared test [χ*^2^*(1, *N* = 101) = 8.33, *p* = 0.004].

To gain insight into whether supporters of each job offer differed in their evaluations of the offers, we conducted a series of mixed-design ANOVAs with job offer preferences as a between-subject factor and offer type as a within-subject factor. In this way, we looked for interaction effects. We present descriptive statistics in Table 1 and the summary of results in Table 2. We found significant main effects of job offer type and interactions of job offer type and preference, but with different patterns depending on the type of evaluations.

For job offer attractiveness, supporters of the SEBUAS offer considered the preferred offer as much more attractive than the WEBAS offer, with a large mean difference of 1.97 [*F*(1,99) = 55.99, *p* < 0.001, $\eta_{\text{p}}^{2}$ = 0.36]. The pattern of results for participants who preferred the WEBAS offer tended to be reversed with a small mean difference of −0.385 [*F*(1,99) = 3.84, *p* < 0.05, $\eta_{\text{p}}^{2}$ = 0.04]. Compared to supporters of the WEBAS offer, supporters of the SEBUAS offer evaluated the SEBUAS offer as much more attractive [*diff* = 1.06 (*F*(1, 99) = 19.45, *p* < 0.001, $\eta_{\text{p}}^{2}$ = 0.16], and considered the WEBAS offer to be much less attractive [diff = −1.30, *F*(1,99) = 32.535, *p*<0.001, $\eta_{\text{p}}^{2}$ = 0.25].

We found a partially different pattern of results for perception of organizational fit. This time, both sets of supporters perceived themselves as significantly better fitted to the SEBUAS job than to the WEBAS job. But again, in the case of supporters of the SEBUAS offer, the significant difference between means was large [*diff* = 2.79, *F*(1,99) = 160.23, *p* < 0.001, $\eta_{\text{p}}^{2}$ = 0.62], and in the case of supporters of the WEBAS offer, the difference between means was smaller, although still significant [*diff* = 0.815, *F*(1,99) = 24.68, *p* < 0.001, partial η^2^ = 0.20)]. Again, compared to supporters of the WEBAS offer, supporters of the SEBUAS offer evaluated the SEBUAS offer as much more attractive [*diff* = 1.00, *F*(1,99) = 22.03, *p* < 0.001, $\eta_{\text{p}}^{2}$ = 0.18], and the WEBAS offer as much less attractive [*diff* = −0.98, *F*(1,99) = 31.21, *p* < 0.001, $\eta_{\text{p}}^{2}$ partial (η2 = 0.24]. Thus, the pattern was similar to the results of the EBE evaluation.

In summary, job offer preferences were related to evaluations of the attractiveness of job offers and salaries, and in the case of supporters of the SEBUAS offer, preferences were positively related with the perception of organizational fit and EBE. Interestingly, supporters of the WEBAS offer expressed a sense of lower organizational fit and perceived the chosen organization as having a weaker organization brand as compared to the rejected offer.

### Materialism and Inclination for S-EB-R as Predictors of Job Offer Preferences

To verify H1a and H2a, regarding the role of materialism and inclination for S-EB-R in job offer preferences, we conducted a logistic regression analysis using the enter method, with two predictors, materialism and inclination for S-EB-R. We present the results in Table 3. Regarding the theoretical assumptions for logistic regression, we found that the predictors in the model explained the preferences better than incidentally [χ^2^(2) = 18,3, *p* < 0.001], and using Hosmer and Lemeshow’s goodness-of-fit statistic, we confirmed that the model was well fit to the data [χ^2^(8) = 4.43, *p* = 0.82]. Both analyzed variables in the model significantly predicted preferences. The direction of beta coefficients showed that participants with a higher level of materialism preferred the WEBAS offer more often, whereas participants with a higher level of inclination for S-EB-R preferred the SEBUAS offer more often. The obtained patterns are inline with H1a and H2a.

**TABLE 3:** Summary of coefficients of the logistic regression model predicting preferred job offer.

	β	*SE*	*p*	95% CI for Exp(B)		
				Lower	Exp(B)	Upper
Constant	0.91 [2.57, 5.32]	1.97	0.65		2.495	
Materialism	0.985 *** [0.49, 1.69]	0.30	0.001	1.515	2.68	4.74
Incl-S-EB-R	−1.25 * [−2.63, −0.34]	0.58	0.01	0.10	0.285	0.83

R^2^ = 0.17 (Cox and Snell)0.23 (Nagelkerke). Model χ^2^(2) = 18.3; *p* < 0.001. A job offer from a strong employer brand with an unattractive salary – 0, a job offer from a weak employer brand with an attractive salary – 1. Incl-S-EB-R – Inclination for self–employer brand relationship. In square brackets, 95% BCa bootstrap confidence intervals based on 1,000 samples. * p < 0.05, *** p < 0.005.

### Materialism and Inclination for S-EB-R as Predictors of Job Offer Evaluations

To understand whether materialism and inclination for S-EB-R may influence various dimensions of offer evaluation, we used a series of hierarchical linear regression analyses, again with two predictors, materialism and inclination for S-EB-R. We present the results in Table 4. Both materialism and inclination for S-EB-R significantly predicted the majority of evaluations of the SEBUAS offer: offer attractiveness, organizational fit, and brand strength. Exceptionally, materialism (but not inclination for S-EB-R) significantly predicted salary attractiveness. In each analysis, the final models explained 24–29% of variance, and so the predictive power of the models was weak or moderate. The directions of signs of beta coefficients indicated that lower materialism and higher inclination for S-EB-R enabled anticipation of more positive evaluations of the SEBUAS offer. The beta coefficients revealed that materialism was a stronger predictor of job offer and salary attractiveness, while the inclination for S-EB-R predicted organizational fit and perception of EBE more strongly. The obtained patterns provide support for H1c and H1e regarding materialism, as well as for H2b and H2d on the role of inclination for S-EB-R; however, we also found that materialism may have impacted perception of EB equity and salary attractiveness, while inclination for S-EB-R predicted brand strength but not salary attractiveness.

**TABLE 4:** Summary of the linear hierarchical model of materialism and inclination for self–employer brand relationship (Incl-S-EB-R) as predictors of offer, brand and salary evaluations.

		Evaluation of SEBUAS offer				Evaluation of WEBAS offer			
		*B*	*SE B*	β	*p*	*B*	*SE B*	β	*p*
		**Attractiveness**				**Attractiveness**			
Step 1	Constant	6.99 [5.98, 7.85]	0.48		0.001	3.14 [1.89, 4.49]	0.61		0.001
	MAT	−0.53 [−0.75, −0.28]	0.115	−0.40****	0.001	0.31 [0.03, 0.55]	0.14	0.23*	0.02
Step 2	Constant	4.20 [2.64, 5.65]	0.89		0.001	3.81 [1.96, 6.05]	0.94		0.001
	MAT	−0.57 [−0.77, −0.36]	0.10	−0.43****	0.001	0.32 [0.03, 0.56]	0.14	0.235*	0.05
	Incl-S-EB-R	0.885 [0.47, 1.38]	0.22	0.36****	0.001	−0.21 [−0.79, 0.21]	0.26	–0.09	0.38
		*R*^2^ = 0.16, *F*(1,98) = 18.63 for step 1; Δ*R*^2^ = 0.13, *F*(2,98) = 19.99 for step 2 (all *p*s < 0.001).				*R*^2^ = 0.05, *F*(1,99) = 5.41, *p* = 0.02 for step 1; Δ*R*^2^ = 0.01, *p* = 0.38, *F(*2,98) = 3.09, *p* = 0.05 for step 2			

		**Perception of organizational fit**				**Perception of organizational fit**			

Step 1	Constant	6.03 [5.12, 6.905]	0.48		0.001	2.72 [1.87, 3.74]	0.50		0.001
	MAT	−0.305 [−0.55, −0.06]	0.12	−0.25**	0.01	0.14 [−0.10, 0.35]	0.11	0.13	0.19
Step 2	Constant	3.12 [1.37, 4.85]	0.97		0.001	3.52 [2.25, 5.29]	0.68		0.001
	MAT	−0.35 [−0.56, −0.13]	0.10	−0.29***	0.002	0.15 [−0.10, 0.37	0.12	0.14	0.15
	Incl-S-EB-R	0.92 [0.43, 1.45]	0.23	0.42****	0.001	−0.25 [−0.65, 0.03]	0.18	–0.14	0.18
		*R*^2^ = 0.06, *F*(1,99) = 6.79, *p* = 0.01 for step 1; Δ*R*^2^ = 0.18, *F*(2,98) = 15.59, *p*s < 0.001 for step 2.				*R*^2^ = 0.02, *F*(1,99) = 1.76, *p* = 0.19 for step 1; Δ*R*^2^ = 0.02, *p* = 0.18, *F*(2,98) = 1.815, p = 0.17 for step 2			

		**EB equity of strong EB**				**EB equity of weak EB**			

Step 1	Constant	6.24 [5.45, 7.06]	0.44		0.001	3.23 [2.34, 4.22]	0.50		0.001
	MAT	−0.27 [−0.49, −0.05]	0.11	−0.27**	0.01	0.10 [−0.13, 0.31]	0.11	0.10	0.315
Step 2	Constant	3.72 [2.52, 4.85]	0.61		0.001	3.58 [2.14, 5.06]	0.72		0.001
	MAT	−0.30 [−0.51, −0.09]	0.10	−0.30****	0.001	0.10 [−0.13, 0.33]	0.11	0.11	0.29
	Incl-S-EB-R	0.80 [0.51, 1.10]	0.14	0.44****	0.001	−0.11 [−0.52, 0.22]	0.19	–0.06	0.53
		*R*^2^ = 0.07, *F*(1,99) = 7.67, *p* = 0.007 for step 1; Δ*R*^2^ = 0.19, *F*(2,98) = 17.77, *p*s < 0.001 for step 2.				*R*^2^ = 0.01, *F*(1,99) = 1.02, *p* = 0.315 for step 1; Δ*R*^2^ = 0.004, *F*(2,98) = 0.70, *p* = 0.45 for step 2			

		**Attractiveness of low salary offered by strong EB**				**Attractiveness of high salary offered by weak EB**			

Step 1	Constant	4.60 [4.03, 5.24]	0.30		0.001	4.62 [4.13, 5.22]	0.26		0.001
	MAT	−0.60 [−0.73, −0.48]	0.07	−0.55****	0.001	−0.04 [−0.17, 0.07]	0.07	–0.06	0.55
Step 2	Constant	5.29 [3.78, 6.89]	0.77		0.001	4.39 [3.13, 6.07]	0.62		0.001
	MAT	−0.59 [−0.72, −0.48]	0.06	−0.54****	0.001	−0.04 [−0.17, 0.08]	0.07	–0.07	0.515
	Incl-S-EB-R	−0.22 [−0.565, 0.17]	0.19	–0.11	0.19	0.07 [−0.34, 0.34]	0.18	0.06	0.526
		*R*^2^ = 0.30, *F*(1,99) = 42.57, *p* = 0.001 for step 1; Δ*R*^2^ = 0.01, *p* = 0.19, *F*(2,98) = 22.30, *p* = 0.001 for step 2.				*R*^2^ = 0.004, *F*(1,99) = 0.37, *p* = 0.55 for step 1; Δ*R*^2^ = 0.004, *p* = 0.53, *F*(2,98) = 0.385, *p* = 0.68 for step 2			

MAT, materialism; Incl-S-EB-R, Inclination for self–employer brand relationship; EB, employer brand; SEBUAS, job offer from a strong employer brand with an unattractive salary; WEBAS, job offer from a weak employer brand with an attractive salary. In square brackets, 95% bias corrected and accelerated confidence intervals reported are reported (confidence intervals and standard errors based on 1000 bootstrap samples). * *p* < 0.05, ** *p* < 0.01, *** *p* < 0.005, **** *p* < 0.001.

Regarding the WEBAS offer, we found only that materialistic values were a significant positive predictor of job offer attractiveness. In particular, participants with a higher level of materialism considered the WEBAS offer more attractive. The obtained patterns are in line with H1b; however, we did not find support for H1d, H2c, or H2e.

In Table 5, we present a summary of the significant hierarchical regression models for anticipated job satisfaction and life satisfaction with materialism and Incl-S-EB-R scores as predictors, separately for supporters of the SEBUAS and WEBAS offer. In the case of anticipated job satisfaction, only the regression model for materialism among supporters of the SEBUAS offer was significant. For SWLS, for supporters of both offers, only materialism was a significant predictor. Thus, we found support for H1g and H1h, but our results were not in line with H1f, H2f, or H2g.

**TABLE 5:** Summary of the linear hierarchical models of materialism and inclination for self–employer brand relationship (Incl-S-EB-R) as predictors of anticipated job satisfaction and overall life satisfaction.

		Supporters of SEBUAS offer				Supporters of WEBAS offer			
		*B*	*SE B*	β	*p*	*B*	*SE B*	*β*	*p*
**Anticipated job satisfaction**									
Step 1	Constant	5.20 [4.22, 6.51]	0.55		0.001	4.07 [2.77, 5.28]	0.62		0.001
	MAT	−0.32 [−0.61, −0.08]	0.14	−0.36*	0.03	-0.09 [−0.35, 0.18]	0.15	–0.105	0.55
Step 2	Constant	5.28 [1.80, 7.18]	1.36		0.001	3.42 [1.77, 5.38]	0.86		0.001
	MAT	−0.32 [−0.62, −0.05]	0.14	−0.36*	0.03	-0.135 [−0.39, 0.17]	0.15	–0.15	0.37
	Incl-S-EB-R	−0.02 [−0.49, 0.83]	0.32	–0.12	0.95	0.255 [−0.17, 0.54]	0.21	0.18	0.22
		*R*^2^ = 0.13, *F*(1,34) = 5.19, *p* = 0.03 for step 1; Δ*R*^2^ < 0.001, *p* = 0.94, *F*(2,33) = 2.52 for step 2 *p* = 0.10.				*R*^2^ = 0.01, *F*(1,63) = 0.70, *p* = 0.41 for step 1; Δ*R*^2^ = 0.03, *p* = 0.16, *F*(2,62) = 1.35, *p* = 0.27 for step 2			
**Overall life satisfaction**									
Step 1	Constant	5.27 [4.29, 6.41]	0.53		0.001	4.45 [3.67, 5.23]	0.39		0.00
	MAT	−0.22 [−0.74, −0.19]	0.14	−0.26*	0.001	−0.23 [−0.40, −0.06]	0.09	−0.26*	0.02
Step 2	Constant	3.91 [−0.09, 5.38]	1.41		0.02	0.3.56 [2.47, 4.66]	0.56		0.00
	MAT	−0.23 [−0.69, −0.19]	0.14	−0.27*	0.001	−0.28 [−0.49, −0.09]	0.10	−0.325*	0.01
	Incl-S-EB-R	0.25 [−0.02, 1.49]	0.35	0.16	0.11	0.35 [−0.06, 0.78]	0.19	–0.25	0.06
		*R*^2^ = 0.20, *F*(1,34) = 8.28, *p* = 0.01 for step 1; Δ*R*^2^ = 0.06, *p* = 0.10, *F*(2,33) = 3.745 for step 2 *p* = 0.01.				*R*^2^ = 0.07, *F*(1,63) = 4.53, *p* = 0.04 for step 1; Δ*R*^2^ = 0.06, *p* = 0.04, *F*(2,62) = 4.51, *p* = 0.015 for step 2			

MAT, Materialism; Incl-S-EB-R, Inclination for self–employer brand relationship. SEBUAS, job offer of a strong employer brand with an unattractive salary; WEBAS, job offer of a weak employer brand with an attractive salary. In square brackets, 95% bias corrected and accelerated confidence intervals reported are reported (confidence intervals and standard errors based on 1000 bootstrap samples). * *p* < 0.05, ** *p* < 0.01, *** *p* < 0.005, **** *p* < 0.001.

### Job-Offer Preferences, Anticipated Job Satisfaction, and Overall Life Satisfaction

To examine whether preferences for job offers were related to anticipated job satisfaction and life satisfaction, we conducted *t*-tests for independent samples. We found a significant difference in anticipated job satisfaction (*t*(99) = 2.34, *p* = 0.02, BCa 95% CI [0.05,0.70]), showing that participants who preferred the SEBUAS offer reported a higher level of anticipated job satisfaction (*M* = 4.06, *SD* = 0.79) than participants who preferred the WEBAS offer (*M* = 3.68, *SD* = 0.77), with a medium-sized effect (*d* = 0.47). In turn, the difference in life satisfaction between participants who favored the SEBUAS offer (*M* = 3.68, *SD* = 0.91) and those who favored the WEBAS offer (*M* = 3.49, *SD* = 0.76), was not significant (*t*(99) = 1.13, *p* = 0.26, BCa 95% CI [−0.15,0.55]), representing a small-sized effect (*d* = 0.23). The obtained pattern of results is in line with Hypothesis 3.

## Discussion

In this study, we examined materialism and psychological employer brand meaning as predictors of job offer preferences, evaluations, and anticipated job satisfaction in a situation of a choice between two job offers. First, satisfactory outcomes for the effectiveness of the experimental manipulations let us conclude that indeed participants perceived the SEBUAS offer in terms of strong EB offering an unattractive salary, and the WEBAS offer as a weak EB offering an attractive salary. Second, each job offer had supporters, with a larger group of supporters for the WEBAS offer than for the SEBUAS offer. This finding is in line with previous studies which show ambiguity in the relative importance of pay and organization characteristics (e.g., Cable and Judge, 1994, pp. 317–348; Powell and Goulet, 1996, pp. 1619–1640; Collins and Stevens, 2002, pp. 685–717; Turban and Cable, 2003, pp. 733–751; Chapman et al., 2005, pp. 928–944; Jones et al., 2006, pp. 167–179; Cheramie et al., 2007, pp. 359–374; Uggerslev et al., 2012, pp. 597–660), a higher importance of pay among business target groups (Cable and Judge, 1996, pp. 294–311; Cheramie et al., 2007, pp. 359–374) and a lower impact of heuristics, such as brands, among experts about given product categories (Maheswaran et al., 1996, pp. 115–133; Ruth, 2001, pp. 99–113). Both effective manipulations and confirmations of previous studies enhance the validity of the new findings.

Regarding the hypothesized relationships, the regression models showed that both materialism and inclination for identity-motives-based S-EB-R were significant predictors of preferences for the SEBUAS offer (negative or positive, respectively), which is in line with H1a and H2a. Both also predicted attractiveness and fit toward the organization for the SEBUAS offer, in expected directions (less positive evaluations in the case of materialism as claimed in H1c and H1e, and more positive evaluations for Incl-S-EB-R, expressed in H2b and H2d). For materialism, results also supported our hypotheses regarding lower anticipated job satisfaction for the SEBUAS offer (H1g), higher attractiveness of the WEBAS job offer (H1b), and lower overall sense of life satisfaction (H1h). Our findings did not reveal support for other hypotheses for the WEBAS offer, regarding the relationships of materialism with fit and anticipated job satisfaction, nor an inclination toward S-EB-R for attractiveness, fit, and anticipated job satisfaction (H1d, H1f, H2c, H2e, and H2g, respectively). Additionally, we confirmed Hypothesis 3, that supporters of the SEBUAS job offer would anticipate higher job satisfaction than supporters of the WEBAS offer.

The overall pattern of results follows a P-O fit approach that the relative importance of instrumental and symbolic job and organization features depends on suitability of personal characteristics and perception of organization features. Additionally, the pattern of findings on materialism extends the results of Cable and Judge (1994, pp. 317–348) on pay attractiveness, by showing that materialism may determine preferences and positive evaluations for attractive salaries, even when organization features were unsatisfactory, and may facilitate rejections and negative evaluations of the attractiveness of job offers with unsatisfactory salaries, even when the organization is perceived as stronger in terms of EB. Our results also follow the findings of Flynn et al. (2011, pp. 5–18) regarding lower brand engagement in self-concept when income is lower (most likely typical for students entering the labor market). Our study introduced a new construct of inclination to S-EB-R as another predictor of attractiveness and perceived fit of an organization having a strong EB. Our findings are in line with theoretical models which emphasize psychological needs satisfaction as constituting a strong brand (e.g., Kapferer, 2008; Keller, 2008; Aaker, 2009) and brands as a means to enhance or express oneself (Park et al., 1986, pp. 135–145; Park et al., 2008; Strizhakova et al., 2008, pp. 82–93; Reimann and Aron, 2009, pp. 65–81). Moreover, our results extend recent findings of Thomas et al. (2017, pp. 508–523) who applied MICT to organizational belonging, as we show the application of MICT to the stage of organization choice.

In a recent study, Unanue et al. (2017b, p. 1755) showed the mediating roles of psychological needs satisfaction and frustration in the relationship between materialism and well-being. Applying this idea to the current results, we posit that a high-salary offer may satisfy the needs of materialists, a low-salary offer may frustrate the needs of materialists, and strong-brand offers may satisfy the needs of individuals with a high inclination for S-EB-R. In turn, the perception of a weak EB may not include content referring to satisfaction or frustration of psychological needs. This reasoning is in line with Keller’s (2008) ideas regarding hierarchical structure of brand knowledge, with more symbolic levels following more concrete levels. The satisfaction of only functional needs without delivering psychological benefits is not enough to feel emotionally connected with a brand. The needs satisfaction-frustration framework and results regarding the different impacts of needs satisfaction and frustration in predicting outcomes for well-being (Unanue et al., 2017b, p. 1755) deliver a valuable interpretation for results on anticipated job satisfaction as well. The preference for the SEBUAS job offer, even though it satisfies identity needs, may be extremely frustrating for materialists, and thus induce a strong affect and evaluative reaction. In turn, the preference for the WEBAS job offer may not have induced a strong enough affect, at least in a short-term perspective, to evoke feeling of lack of fit or dissatisfaction. Despite higher anticipated job satisfaction among supporters of the SEBUAS than the WEBAS job offer, the inclination for S-EB-R was not a significant predictor of anticipated job satisfaction. It could be that only having an actual sense that the organization satisfies one’s psychological identity needs induces the feeling of job and life satisfaction (Styśko-Kunkowska, 2017, pp. 65–84).

Our results on the differences in anticipated job satisfaction between two sets of job offer supporters complement the findings of Kuvaas et al. (2017, pp. 244–258) regarding the relationships of intrinsic and extrinsic (including salary) motivation with positive and negative outcomes. We showed that despite preference and positive job offer evaluation for the extrinsically motivated WEBAS offer, individuals anticipated less satisfaction from the moment of offer choice. Considering the relationship of job satisfaction with overall well-being (Sousa-Poza and Sousa-Poza, 2000, pp. 517–538; Wright and Cropanzano, 2000, pp. 84–94) and personal and work outcomes (Harter et al., 2003, pp. 205–224), this preliminary belief may have personal and professional negative outcomes in the future. Drawing from recent findings on the framework of von Walter et al. (2012, pp. 116–135) regarding the relationship of P-O fit, abstract-detailed level of processing, near versus distant time perspectives, and organization attractiveness, we posit a brand-based preference for job offers may be both abstract and related to holding a future perspective, including the anticipation of higher job satisfaction. In contrast, a salary-based preference is concrete and focused on a near-time perspective, so it may facilitate omitting future job satisfaction in the moment of making the decision. The presented interpretative mechanisms require further investigation.

### Limitations and Future Studies

Our study delivers new, interesting insights within the field of organizational and business psychology on the roles of pay and EB in decision processes. Nevertheless, we noticed several limitations. We restricted our sample to the business and finances sectors to replicate previous findings (Cable and Judge, 1994, pp. 317–348; Lievens and Highhouse, 2003, pp. 75–102); however, future studies may benefit from research with different target groups and EBs. We used an explicit measure of psychological EB meaning following considerations that, due to reflexivity and high motivation in the process of job choice, explicit attitudes may be good predictors of actions, similar to research on brands (Maheswaran et al., 1992, pp. 317–336) and in the political domain (Friese et al., 2007, pp. 246–255). Following the possible discrepancy between implicit measures and self-reporting in both identity motives (Vignoles, 2011, pp. 403–432) and consumer behavior domains (Perkins et al., 2008, pp. 461–475), we find this valuable for further examination. Furthermore, considering the complex relationships of individual dispositions and pay policies (Cable and Judge, 1994, pp. 317–348), the role of compensation structures other than salary merit further investigation as well.

### Implications

Despite the limitations, our study delivers promising implications both for theory and practice development, offering new and interesting ideas for both academics and practitioners. In the previous literature, symbolic EB meaning was examined mainly in the context of particular brands and in evaluation of employer attractiveness; despite interest in the consumer brand area (Strizhakova et al., 2008, pp. 82–93; Sprott et al., 2009, pp. 92–104; Fischer et al., 2010, pp. 823–839; Puligadda et al., 2012, pp. 115–130), the idea of overall individual differences in EB meaning was not discussed. We introduced the new psychological construct of inclination for self–employer brand relationship based on identity motives and showed that it may have important implications for the job decision process. Our study also adds to the literature concerning P-O fit and job satisfaction, by showing that people may prefer one organization over another despite lower perceived organizational fit and anticipation of less job satisfaction in the future.

For practitioners, we emphasize the importance of recognition of the perception of EB and salary attractiveness in a target group, and that strategies juxtaposing strong/weak EBE and low/high salary may convince individuals with different psychological needs to accept one or the another job proposal. Regarding possible distant consequences for efficiency and employee life quality (Harter et al., 2003, pp. 205–224; Kristof-Brown et al., 2005, pp. 281–342), we highlight that a sense of organizational fit and job satisfaction is formed at the moment of job offer acceptance. Overall, we suggest that understanding determinants of job offer preferences may extend research into new fields and result in increased organizational fit and higher individual job well-being via relevant employer branding procedures.

## Data Availability Statement

The raw data supporting the conclusions of this manuscript will be made available by the authors, without undue reservation, to any qualified researcher.

## Ethics Statement

The studies involving human participants were reviewed and approved by Committee of Ethics in Research of the Faculty of Psychology, University of Warsaw. Written informed consent for participation was not required for this study in accordance with the national legislation and the institutional requirements.

## Author Contributions

MS-K and ZK contributed to the design of the study, conducted the statistical analysis, and reviewed and approved the final manuscript. MS-K developed the initial conceptual framework and preliminary design of the study. MS-K wrote and edited the manuscript with input from ZK. ZK prepared the Internet forms, with input from MS-K, and organized and controlled the process of data gathering.

## Conflict of Interest

The authors declare that the research was conducted in the absence of any commercial or financial relationships that could be construed as a potential conflict of interest.
