# Supplementary material for: Choice Between Salary and Employer Brand: The Roles of Materialism and Inclination to Develop an Identity-Motives-Based Relationship With an Employer Brand
Source: Front Psychol. 2020 Mar 27;11:555. doi: 10.3389/fpsyg.2020.00555 (PMC7118199; doi:10.3389/fpsyg.2020.00555)
Supplement: Supplementary file 1 [file Table_1.DOCX]

Supplementary Material

# Aims of the Pilot Study

The aims of the pilot study were to choose the proper quotas of attractive and unattractive salaries, names of strong and weak employer brands (EBs), and names of job positions, to develop manipulations and stimulus materials.

# Method

## Participants

In the study, 52 university students representing ten public and private universities (71.2% women) in the fields of management, economics, and mathematical sciences participated. They were between 19 and 26 years of age. Forty-six participants reported having work experience with at least one employer; 44.2% reported they worked currently, and 15.4% were planning to search for a job within the next three months.

## Procedure

We distributed aComputer-Assisted Web Interview on the LimeSurvey platform to students of management, economics, and mathematical sciences. After screening questions about current educational status and field of study, we asked participants to imagine that, after graduation, they received a full-time offer for an attractive job position and to answer open- and closed-ended questions on salary level, job position, and employer brand (including spontaneous employer brand preferences), along with ranking companies in a list provided to them according to their desire to work for each company.

# Results

The students spontaneously mentioned 31 brand names as their preferred places of employment and 14 brand names as undesirable employers. For example, Google was on the spontaneous list of preferred EBs six times and never on the list of undesirable EBs, while McDonald’s was on the list of undesirable EBs 17 times and never on the list of preferred EBs. In the ranking of provided EBs, Google obtained the best position most often and had the lowest mean (*M* = 2.35, *SD* = 1.74, *Mo* = 1, *Me* = 2, *Minimum* = 1, *Maximum* = 8; where 1 means the most attractive) when compared to the other nine EB names. In turn, the mean for McDonald’s was highest and most often in last position (*M* = 7.23, *SD* = 1.98, *Mo* = 9, *Me* = 7.50), and this EB was never placed in the best position (*Minimum* = 2, *Maximum* = 8). Google also obtained the highest mean in the EB equity questionnaire (*M* = 4.24 and *SD* = 0.62, on a scale where 5 indicated strong EB), while McDonald’s was among five brands with the lowest means (*M* = 2.46, *SD* = 0.86). Google and McDonald’s differed significantly in EB equity evaluations in a *t*-test for dependent samples (*t*(51) = 13.89, *p*<. 001).

Regarding spontaneous answers about salaries for a preferred job position, we found that acceptable levels of gross salary varied from 3,000 to 4,500 Polish złoty. A comparison was made of supported answers for six salary levels (2,500, 3,500, 4,500, 5,500, 6,500, 7,500) in terms of attractiveness and plausibility or realisticness, with the highest anchor indicating the most realistic and the most attractive on a 7-point scale.As predicted, students perceived the higher salary levels as more attractive and less realistic. Among the lowest salaries, only 2,500 in local currency was perceived as both realistic (*M* = 5.94, *SD* = 1.56) and unattractive (*M* = 2.29, *SD* = 1.35, *Minimum* = 1, *Maximum* = 5). A salary of 5,500 was perceived as rather attractive (*M* = 5.98, *SD* = 0.80, *Minimum* = 4, *Maximum* = 7) and moderately realistic (*M* = 3.5, *SD* = 1.45), while 6,500 was perceived as highly attractive (*M* = 6.42, *SD* =0.57, *Minimum* = 5, *Maximum* = 7), but rather unrealistic (*M* = 3.12, *SD* = 1.45).

After grouping the spontaneous answers on job position by field (i.e., controller, analyst, accountant), we found that accountant appeared most often, and that senior positions were mentioned more often than junior ones. From a supported list of eight job positions (including six junior positions for analyst, accountant, financial controller, PR manager, audit consultant, media planner, marketing specialist, and digital marketing specialist), students preferred the junior financial controller (*fre* = 20) and younger analyst positions (*fre* = 17) most often; however, 19 participants replied with the answer “none of them.” Cross tabulations revealed that younger controller in the aided list was chosen by participants who indicated previously they preferred senior positions, while the younger analyst position was chosen by participants who preferred junior positions. Participants who spontaneously mentioned the position of accountant chose the controller positions from the list.

**4 Discussion**

The results of the pilot study indicate that within a target group of Polish students in the fields of management, economics, and mathematical sciences, compared to other tested employer brands, one may treat Google as a strong and McDonald’s as a weak employer brand.

The results for the salary levels showed that a salary of 2,500 was perceived both as realistic and unattractive by the majority of participants, 6500 was attractive but rather unrealistic, and 5,500 was on the border of being realistic. To avoid a ceiling effect and to stay close to reality, we chose 6,000 as an attractive offer.

The results for job positions showed a variety of preferences, but they also suggested some directions. We decided the position of financial controller, without any prefix such as senior or junior, would encourage both people interested in accounting and those interested in senior positions.
